# Timely initiation of postpartum contraceptive utilization and associated factors among women of child bearing age in Aroressa District, Southern Ethiopia: a community based cross-sectional study

**DOI:** 10.1186/s12889-018-5981-9

**Published:** 2018-09-06

**Authors:** Aregahegn Dona, Muluemebet Abera, Tsedach Alemu, Dawit Hawaria

**Affiliations:** 1 Aroressa District Health Office, Aroressa, Majo, Ethiopia; 2 Population and Family Health Department, Institute of Health, Jimma University, Jimma, Ethiopia; 3 Yirgalem Hospital Medical College, Yirgalem, Ethiopia

**Keywords:** Timely initiation, Postpartum contraceptives, Aroressa

## Abstract

**Background:**

Globally, more than 90% of women during the first year of postpartum period want to either delay or avoid future pregnancies. The first year postpartum period is more crucial time to use modern contraceptives that enhance maternal and child health, so more attention should be given on time of initiating modern contraceptive utilization after delivery. Therefore, the aim of this study was to assess the magnitude and associated factors of timely initiation of postpartum contraceptive utilization among women of child bearing age in Aroressa district, Southern Ethiopia.

**Methods:**

The study was conducted in Aroressa district from March 15 to April 15, 2017. A community based cross-sectional study design with interviewer administered structured and pretested questionnaire was used. Multistage sampling technique was employed involving a total of 695 women of child bearing age who delivered a child in the past 12 months prior to the study period. Data were cleaned, coded and entered into Epi data version 3.1, then exported to statistical package for social science version 20 for analysis. Descriptive statistics, Bivariate and Multivariate logistic regression analysis were done. *p*-value < 0.05 was used to consider significant variables.

**Results:**

The magnitude of timely initiation of postpartum contraceptive utilization was found to be 31.7% [95% CI (28, 36)]. Antenatal care [AOR = 1.94, 95% CI (1.23, 3.01)], postnatal care [AOR = 1.90, 95%CI (1.23, 2.94)], spousal communication on contraceptive methods [AOR = 1.63, 95% CI (1.09, 2.41)] and resumption of menses after delivery [AOR = 2.6, 95% CI (1.47, 3.81)] were predictors positively associated with timely initiation of postpartum contraceptive utilization.

**Conclusion:**

The magnitude of timely initiation of postpartum contraceptive utilization was low. Strengthening integration of family planning information with antenatal and postnatal care follow up and encouraging spousal communication by promoting information, education and communication activities is important to enhance contraceptive use on timely manner.

## Background

Globally, Family Planning is recognized as a key life-saving intervention for mothers and their children [1]. It can avert more than 30% of maternal deaths and 10% of child mortality if couples space their pregnancies more than 2 years apart [2]. Postpartum family planning has an important role in strategies to support longer birth intervals or reduce unintended pregnancy and its consequences [1].

Globally, more than 90% of women during the first year of postpartum period want to either delay or avoid future pregnancies. However, in most cases, sexual activity is resumed without using any contraceptive method [3].

Unintended pregnancies particularly among women in developing countries and poor individuals are linked to elevated health problems that resulted in high number of maternal and neonatal deaths [4, 5]. An estimated 30 million unplanned births and 40 million abortions, half of them illegal and unsafe, occurred annually in low- and middle-income countries [6]. Maternal deaths can be declined by 7–35% as the number of children per woman decreased, however, pregnancy can occur within 45 days of postpartum period if woman didn’t use any modern contraceptive method [3, 7].

The highest rate of mistimed pregnancy become a big problem especially in sub-Sahara Africa where approximately half of all pregnancies were reported to have come soon that could have been prevented with increased access to effective utilization of modern postpartum contraceptive methods on timely manner [8].

In Ethiopia, a national survey of 2013 stated that the prevalence of unintended pregnancy was 24% [9]. In addition Ethiopia Demographic and Health Survey of 2011 showed that about 16% of pregnancies were mistimed and 9% was reported as unwanted pregnancy [10, 11].

The first year of postpartum period is a crucial time to use modern contraceptive methods on right time to prevent unwanted pregnancy, however, many women do not realize that they are at a risk of becoming pregnant during this period [12]. Study has shown that children born too close to previous birth, especially if the interval between the births is less than 2years, are at increased risk of morbidity and mortality that can easily be prevented by using modern contraceptive methods on right time after delivery [13, 14]. Another Study has also revealed that for the most of women, the average time to first postpartum ovulation is 45 days and can occur as early as 25 postpartum days [15]. An analysis of data from Demographic and Health Survey of 17 developing countries also found that an average of 25% of couples who wanted to space or limit their children were not using any form of modern contraceptive method on right time after delivery [16].

According to Ethiopia Min Demographic and Health Survey 2014, the most of women were not utilizing modern contraceptive after giving birth as a result of missing opportunities for early utilization of postnatal care [17]. It was also found that nearly 29% of current pregnancies were reported as either mistimed or unwanted that would easily be prevented if the women were using modern contraceptive methods on timely manner after delivery [18].

The Ethiopia Federal Minister of Health has developed Family Planning guideline with close consideration of and reference to the Ethiopian Constitution, scientific evidence, as well as international declarations and conventions. Modern Family Planning methods like intrauterine contraceptive device, hormonal, barrier and surgical methods are available in Ethiopia which are provided by the skilled health workers through the modalities of facility-based, community-based, social marketing and outreach services at government and private health institutions based on this guideline [19].

Despite a sound positive impact of initiating post-partum contraceptive utilization on right time to improve maternal and child health, in Ethiopia less attention has been given to the time of initiating it and little was known about the practices and factors affecting utilization of this service on timely manner. Therefore, the aim of this study was to assess the magnitude of timely initiation of postpartum contraceptive utilization and associated factors among women of child bearing age in Aroressa district, Southern Ethiopia. In addition, this study can help policy makers and other stakeholders to improve up take of family planning services in the community through taking right actions on identified factors.

## Methods

### Study area and period

Aroressa district is one of 23 districts in Sidama zone, Southern Ethiopia which found at 181 km and 454 km apart from Hawassa and Addis Ababa City respectively. It has 30 rural and 3 urban Kebeles with a total population of 215,399. From this about 49.8% are females. The women of reproductive age group account 23.3% of the total population and the total number of estimated delivery in 2016/17 was 7453. It has one primary hospital, 8 health centers, 33 health posts, 3 primary private clinics and 4 private drug stores [20]. The study was conducted from March 15–April 15, 2017.

### Study design and sample size determination

A community based cross-sectional study design was used. The sample size was determined by using single population proportion formula considering the following assumptions: 95% confidence level, proportion of 0.37 [21], margin of error 5%, design effect 2 and estimated non response rate of 10%. Accordingly, total sample size calculated was 695.

### Sampling procedure and study population

A multistage sampling technique was used to identify study subjects. First Kebeles (smallest administrative units of Ethiopia) were stratified into urban and rural Kebeles. Then ten rural and one urban Kebeles were randomly selected from total Kebeles in the district. Censes was done to get all lists of the eligible women prior to the study period and identification number was given for each household with eligible women. Sampling frame was developed for each selected kebele separately based on result of censes. Then calculated sample size was proportionally allocated to each selected Kebele based on its total number of eligible women and finally study subjects were selected by using simple random sampling technique. Incase when there were two or more eligible women in the same household, lottery method was applied to select one of them. Source Population was all women of child bearing age who gave birth in the past 12 months prior to the study period in the Aroressa district, Southern Ethiopia. Randomly selected women who fulfilled inclusion criteria were considered as Study Population. The women who were above 42 days of postpartum and lived at least for 6 months in the selected Kebeles have been included in the study, but those who were sick and unable to respond during study period were excluded.

### Data collection tool and procedure

Structured questionnaire adapted from related literatures was used after some modification to make it consistent with the objective of the study and conceptual frame work. Data were collected by using interviewer administered a structured and pretested questionnaire that contain socio-demographic, economic, and socio-cultural factors, items related to knowledge and attitudes on benefits of using modern contraceptive methods, maternal health services utilization and obstetric characteristics of the study subjects. Data were collected by ten data collectors who have completed grade ten and had previous experience of data collection. Two health professionals (Bachelor degree) were recruited as supervisors. To assure data quality, properly designed data collection tool was developed in English after revising related literatures and translated in to local language (Sidaamu Afoo) and back to English by language experts to check its consistency. Ten data collectors and two supervisors who can read and speak local language fluently were trained for 2 days by principal investigator before starting actual data collection. Training was given on general objective of the study, contents of the tool, how to approach the study participants and keep their confidentiality. Before starting actual data collection, the tool was pre-tested on 5% (35 women) of the sample in Kechawo kebele which was out of the selected Kebeles. Collected data were checked for completeness and consistency by supervisors and principal investigator at the end of each day. To reduce non-response rate, appropriate time was adjusted for repeated visits when the respondents were unavailable. Double data entry was applied to minimize data entry error. Dependent variable was timely initiation of postpartum contraceptive utilization and independent variables were Socio-demographic, economic and Socio-cultural factors, knowledge and attitude on benefits of using modern contraceptive methods, use of maternal health service, and Obstetric factors.

### Data analysis

After completing data collection, the data were cleaned, coded and enter into Epi data version 3.1. Then exported to statistical package for social science (SPSS) version 20 and checked for missing values before analysis. Descriptive analysis was done for each predictor variables. Cross tabulation was also performed to see the distribution of different variables in relation to outcome variable. Principal component analysis was done for the items used to measure attitudes of the respondents. Multi-collinearity among independent variables was checked. The goodness-of-fit of the model was also checked by Hosmer-Lemshow goodness of model fit. Bivariate analysis was done for each independent variable with outcome variable and variables that were associated with outcome variable at *p*-value ≤ 0.25 were considered as candidates for multivariate logistic regression and finally entered into multivariate logistic regression model to control possible confounders and get final model. Backward stepwise logistic regression was used to identify variables which had the largest contribution to the model. Adjusted odds ratio (AOR) with 95% confidence interval (CI) was calculated to determine the presence and strength of association among predictors and outcome variables. *p*-value < 0.05 was used to consider significant variables. Results were described by texts, tables and figures.

### Operational definitions

Timely initiation of postpartum contraceptive utilization: Starting of modern contraceptive utilization within 6 weeks of post-partum period.

Knowledgeable: If the respondent answered mean score and above from knowledge related questions on benefits of modern contraceptive methods.

Have positive attitude: Those who scored mean and above for attitude related items.

## Results

### Socio-demographic and economic factors

From 695 study participants planned for interview, about 684 respondents were interviewed making a response rate of 98.4%. The mean age of the respondents was 25.4 (SD ±5.1) with the minimum and the maximum age of 17 and 41 years respectively. Majorities (89.5%) of the respondents were married and about 78.9% were followers of protestant. Regarding educational status of the respondents, 48.4% had no formal education (Illiterate). Majority (92.3%) of the respondents were from Sidama ethnicity group (Table 1).

**Table 1:** Socio-demographic and economic characteristics of the respondents in Aroressa District, Southern Ethiopia, 2017

Variables and categories (*n* = 684)	Frequency	Percent (%)
Age of the respondents		
15–19	76	11.1
20–24	264	38.6
> =25	344	50.3
Marital status of the respondents		
Single	22	3.2
Married	612	89.5
Separated /Widowed	50	7.3
Religion of the respondents		
Protestant	540	78.9
Orthodox	64	9.4
Muslim	45	6.6
Catholic	35	5.1
Educational status of the respondents		
Illiterate	331	48.4
Literate	353	51.6
Occupational status of the respondents		
Housewife	249	36.4
Merchant	156	22.8
Student	204	29.8
Government employee	75	11.0
Residence of the respondents		
Rural	508	74.3
Urban	176	25.7
Ethnicity of the respondents		
Sidama	631	92.3
Others^a^	53	7.7
Income		
< 500 Birr	345	50.4
> =500Birr	339	49.6

^a^Amhara, Wolaita, Oromo, Gurage

### Socio-cultural characteristics of the respondents

Regarding Socio-cultural characteristics of the respondents, 310 (45.3%) of them have discussed with their husband on number of the children they desired to have in the future. From those who have discussed with their husband, majority (70.1%) of them decided together. More than half (58%) of the respondents have discussed with their husband on modern contraceptive utilization and about 56.3% of them decided together. In terms of husband’s approval on modern contraceptive utilization, 456 (66.7%) of the respondents reported that their husbands support them to utilize modern contraceptive methods.

### Knowledge and attitude of the respondents on benefits of using modern contraceptive methods

Almost all (99.7%) of the respondents heard about modern contraceptives methods and more than half (56.1%) of them were knowledgeable on benefits of modern contraceptive utilization.

In relation to the attitudes of the respondents towards the benefits of contraceptive utilization more than half (52.6%) of them had negative attitudes (Table 2).

**Table 2:** Attitudes of the respondents towards the benefits of using modern contraceptives in Aroressa District, Southern Ethiopia, 2017

Variable	Strongly disagree	Disagree	Neutral	Agree	Strongly agree
Contraceptive utilization is beneficial for your health	161(23.5)	182(26.6)	149(22)	167(24.3)	25(3.6)
Contraceptive utilization can make you strong during pregnancy	196(28.7)	161(23.5)	155(23)	153(22.4)	19(2.4)
Contraceptive utilization can help you during time of delivery	173(25.3)	168(24.6)	271(40)	61(8.9)	11(1.2)
Use of modern contraceptives can help you to live a good life	92(13.5)	99(14.5)	465(68)	16(2.2)	12(1.8)
Use of modern contraceptives can improve the health of your child	54(7.9)	192(28)	84(12.2)	329(48.2)	25(3.7)
Use of modern contraceptives is good for growth of your baby	411(60.1)	155(22.7)	105(15.3)	11(1.6)	2(0.3)
Attitude of the respondents	Negative attitude			360	52.6%
	Positive attitude			324	47.4%

### Characteristics of the respondents related to maternal health service utilization

Regarding to the respondents’ maternal health service utilization characteristics, about 419 (61.3%) of them had previous history of using modern contraceptive methods before last pregnancy and nearly half (47.5%) have attended antenatal care during their last pregnancy. Four hundred seventy (68.7%) gave their last birth at home. Concerning postnatal care service utilization only 66.2% of the respondents attended postnatal clinic after their last delivery (Table 3).

**Table 3:** Characteristics of the respondents related to maternal health service utilization in Aroressa District, Southern Ethiopia, 2017

Variables and categories	Frequency	Percent
Used modern contraceptives before last pregnancy (*n* = 684)		
No	265	38.7
Yes	419	61.3
Attended antenatal clinic during last pregnancy (*n* = 684)		
No	325	47.5
Yes	359	52.5
Number of antenatal care visit during last pregnancy (*n* = 359)		
1	141	39.3
2–3	106	29.5
> =4	112	31.2
Got advice from health professionals about postnatal care during ANC follow up (*n* = 359)		
No	123	34.3
Yes	236	65.7
Place of delivery (*n* = 684)		
Home	470	68.7
Health institution	214	31.3
Have postnatal care after last delivery (*n* = 684)		
No	231	33.8
Yes	453	66.2
Time of starting postpartum contraceptives (*n* = 684)		
< = 6 weeks postpartum	217	31.7
> 6 weeks postpartum	467	68.3

### Reproductive characteristics of the respondents

In terms of the reproductive characteristics, about 60.2% of them were above 6 months postpartum, 41.7% had history of two to three pregnancies and from those who had experience of two and above pregnancies, about 51.6% delivered their last child within two to 3 years after previous birth. Nearly half (48.7%) of them had two to three living children and 488 (71.4%) have seen menses after their last delivery.

### Magnitude of timely initiation of postpartum contraceptive utilization

**Fig. 1:**
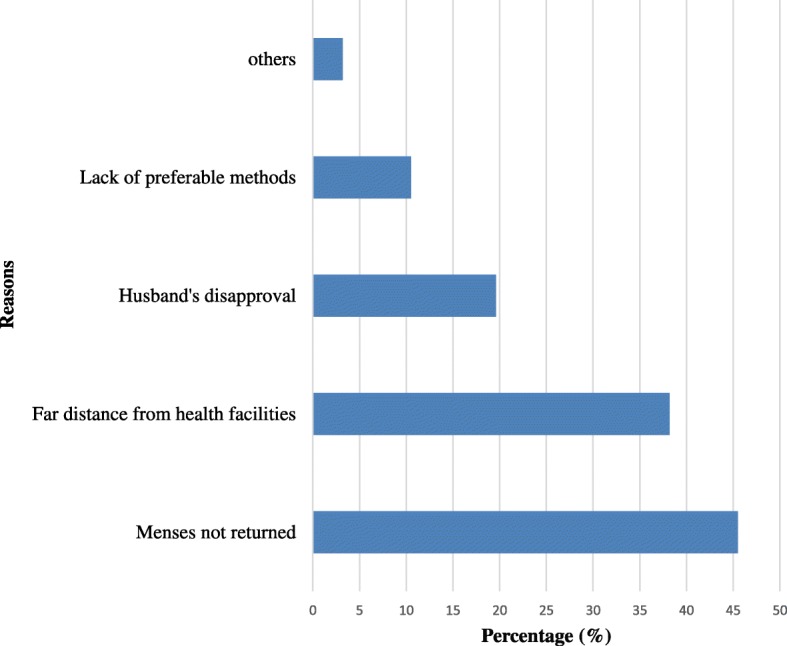
Reasons for not initiating postpartum contraceptive utilization on time in Aroressa District, Southern Ethiopia, 2017

**Fig. 2:**
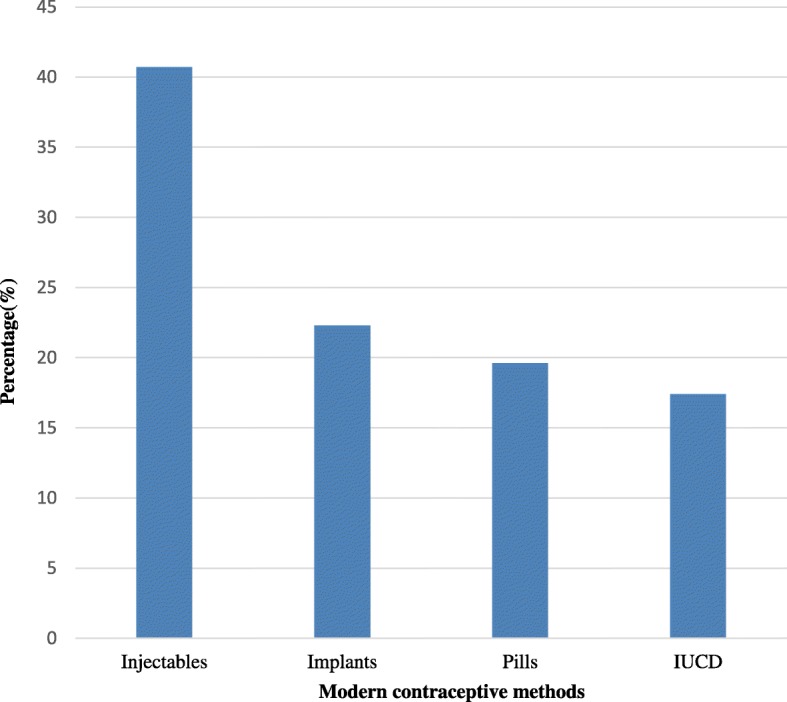
Prevalence of modern contraceptive methods utilized by respondents in Aroressa District, Southern Ethiopia, 2017

Regarding characteristics of the respondents related to timely initiation of postpartum contraceptive utilization, about 217 (31.7%) of the total study participants have initiated postpartum contraceptive utilization on recommended time. The reason for not initiating postpartum contraceptive utilization on recommended time were far distance from health facility, husband’s disapproval, lack of preferable methods and others (Fig. 1). In relation to the choice of contraceptive methods, majority of the contraceptive users were using Injectables (40.7%) followed by Implants (22.3%) (Fig. 2).

### Factors associated with timely initiation of postpartum contraceptive utilization

In bivariate analysis educational status of the mother, spousal communication on contraceptive method, attitude towards contraceptive methods, antenatal care follow up, place of delivery, postnatal care and resumption of menses were associated with timely initiation of postpartum contraceptive utilization. In multivariate logistic regression analysis attending antenatal care, postnatal care, spousal communication on contraceptive methods and resumption of menses were significantly associated with timely initiation of postpartum contraceptive utilization (Table 4).

**Table 4:** Crude and adjusted odds ratio (OR) and 95% confidence interval (CI) of factors associated with timely initiation of postpartum contraceptive utilization in Aroressa district, Southern Ethiopia, 2017

Variables and categories	Initiated postpartum contraceptive utilization on time		COR (95%CI)	AOR (95% CI)
	Yes	No		
Educational status of the mother				
Illiterate	121	212	1	
Literate	96	255	0.28(0.15, 0.51)*	0.6(0.29, 1.27)
Discuss with husband on contraceptive methods				
No	65	223	1	
Yes	152	244	2.4(1.7, 3.5)*	1.63(1.09, 2.4)**
Attitudes towards contraceptive utilization				
Negative	112	248		1
Positive	105	219	0.31(0.13, 0.51)*	0.9(0.55, 1.41)
Had ANC during last pregnancy				
No	40	188	1	
Yes	177	279	2.8(1.87, 4.29)*	1.94(1.2, 3.0)**
Place of delivery				
Home	165	305	1	
Health institution	52	162	0.7(0.45, 0.99)*	1.2(0.8, 1.91)
Attended postnatal care				
No	42	142	1	
Yes	175	325	1.97(1.3, 2.97)*	1.9(1.23, 2.9)*
Menses returned after last delivery				
No	31	169	1	
Yes	186	298	3.4(2.2, 5.2)**	2.6(1.47, 3.8)**

* = statistically significant at *p*-value < 0.05, ** = significant at *p*-value < 0.001

Concerning utilization of maternal health services, women who have attended antenatal care during their last pregnancy were 1.94 times [AOR = 1.94, 95% CI: (1.21, 3.03)] more likely to initiate postpartum contraceptive utilization on time than those who have never attended antenatal care follow up during their last pregnancy. Women who have attended postnatal care after their last delivery were 1.91 times [AOR = 1.91, 95% CI: (1.23, 2.94) more likely to initiate postpartum contraceptive utilization on time than those who have never attended postnatal care after their last delivery.

Women who have discussed with their partners on contraceptive methods were 1.63 times [AOR = 1.63, 95% CI: (1.09, 2.41)] more likely to initiate postpartum contraceptive utilization on time than those who have never discussed.

In relation to reproductive characteristics of the respondents women whose menses was returned after last delivery were 2.6 times [AOR = 2.6, 95% CI: (1.47, 3.81)] more likely to initiate postpartum contraceptive utilization on time than those who haven’t seen menses after their last delivery.

## Discussion

Postpartum family planning is crucial to increase birth intervals and reduce unintended pregnancy including its consequences. It can prevent maternal and child mortality if women start its utilization as early as possible after delivery [1]. This study has attempted to identify the magnitude of timely initiation of postpartum contraceptive utilization and associated factors among women of child bearing age in Aroressa district, Southern Ethiopia. Accordingly the magnitude of timely initiation of postpartum contraceptive utilization was found to be 31.7% [95% CI (28, 36)]. This finding is consistent with similar studies done in Malawi and Kenya [22, 23]. However, this finding was higher than the findings of similar studies previously done in Ethiopia [24, 25]. It was also higher than study done in Nigeria [26]. This difference might be due to improvement in health service delivery, difference in study period as well as socio-economic status of the study participants. However, this finding was found to be lower when compared with study done in Northern Ethiopia and India [21, 27]. The possible explanation for this variation might be difference in study period, socio-economic and socio-cultural status as well as availability and accessibility of the health services.

The present study revealed that women who have attended antenatal care during their last pregnancy were 1.94 times [AOR = 1.94, 95% CI: (1.23, 3.01)] more likely to initiate postpartum contraceptive utilization on time than those who have never attended antenatal care follow up during their last pregnancy. This finding is in line with other similar studies done in Nigeria, south East Asia, Nepal and Ethiopia [26, 28–31]. The possible explanation might be women who attended antenatal care clinic during pregnancy may have more information on benefits of initiating postpartum contraceptive utilization on timely manner and this can increase their intention to use it on time after delivery.

Postpartum women who have attended postnatal care after their last delivery were 1.91 times [AOR = 1.91, 95% CI: (1.23, 2.94) more likely to initiate postpartum contraceptive utilization on time than those who have never attended postnatal care after their last delivery. This finding is consistent with other similar studies done in Nigeria and Ethiopia [25, 26, 29, 32, 33]. This might be due to that postnatal care visit give the opportunity of getting more information and counselling from health professionals and can help postpartum women to use contraceptive methods on effective and timely manner [34].

Postpartum women who have discussed with their husbands on contraceptive methods were 1.63 times [AOR = 1.63, 95% CI: (1.09, 2.41)] more likely to initiate postpartum contraceptive utilization on time than those who have never discussed. The possible explanation for this might be that women can get more information and support to utilize maternal health services through discussing with their spouses, so this might increase their intention to access contraceptive methods in efficient and timely manner after delivery. This is also supported by other studies [24, 35–38].

Women whose menses was returned after last delivery were 2.6 times [AOR = 2.6, 95% CI: (1.47, 3.81)] more likely to initiate postpartum contraceptive utilization on time than those who haven’t seen menses after their last delivery. This finding is also supported by other studies done in Ethiopia [21, 24, 33]. This might be explained by the fact that postpartum women whose menses is returned after delivery may assume that they are at risk of getting pregnancy, so this can initiate them to start postpartum contraceptive utilization on timely manner.

This study has strength in assessing important public health issue which can play a great role of improving maternal and child health specially in resource limited areas. However, it has some limitations: it mainly focused on individual level factors, but factors related with health system and health care providers are not addressed.

## Conclusions

The magnitude of timely initiation of postpartum contraceptive utilization was found to be low in the study area. Having antenatal and postnatal care, spousal communication and resumption of menses after last delivery were factors positively associated with timely initiation of postpartum contraceptive utilization.

Health care providers should give family planning counselling during antenatal and immediate post-partum period with the intention of informing, motivating and educating women to make voluntary and informed choices about post-partum contraceptive use on timely manner. Aroressa District Health Office should encourage dissemination of information, education and communication activities to strength integration of Family Planning information with antenatal and postnatal care as well as to encourage spousal communication on contraceptive methods to reach all eligible women.
